# Cholesteryl ester transfer protein (CETP), HDL capacity of receiving cholesterol and status of inflammatory cytokines in patients with severe heart failure

**DOI:** 10.1186/s12944-018-0888-0

**Published:** 2018-10-20

**Authors:** Ana Elisa M. Martinelli, Raul C. Maranhão, Priscila O. Carvalho, Fatima R. Freitas, Bruna M. O. Silva, Milena N. C. Curiati, Roberto Kalil Filho, Antonio Carlos Pereira-Barretto

**Affiliations:** 1 Laboratorio de Metabolismo e Lipides, Instituto do Coracao, Hospital das Clinicas HCFMUSP, Faculdade de Medicina, Universidade de Sao Paulo, Av. Dr. Enéas de Carvalho Aguiar, 44. 1° subsolo, São Paulo, SP 05403-000 Brazil; 2 Faculdade de Ciencias Farmaceuticas, Universidade de Sao Paulo, São Paulo, Brazil; 3 Hospital Santa Marcelina, São Paulo, Brazil; 4 Servico de Prevencao e Reabilitacao Cardiovascular, Instituto do Coracao, Hospital das Clinicas HCFMUSP, Faculdade de Medicina, Universidade de Sao Paulo, Sao Paulo, Brazil

**Keywords:** Heart failure progression, Lipid transfer, Lipoproteins, Cholesteryl ester transfer protein (CETP), Lipid nanoparticles

## Abstract

**Background:**

Heart failure (HF) courses with chronic inflammatory process and alterations in lipid metabolism may aggravate the disease. The aim was to test whether the severity of HF, using brain natriuretic peptide (BNP) as a marker, is associated with alterations in functional aspects of HDL, such as lipid transfer, cholesterol ester transfer protein (CETP) and lecithin-cholesterol acyltransferase (LCAT) concentration.

**Methods:**

Twenty-five HF patients in NYHA class I/II and 23 in class III/IV were enrolled. Plasma lipids, apolipoproteins, CETP, LCAT, oxidized-LDL (oxLDL) and paraoxonase-1 (PON-1) activity were determined. Lipid transfer from a donor artificial nanoparticle to HDL was measured by in vitro assay.

**Results:**

Total cholesterol (*p* = 0.049), LDL-C (*p* = 0.023), non-HDL-C (*p* = 0.029) and CETP, that promotes lipid transfer among lipoproteins (*p* = 0.013), were lower in III/IV than in I/II group. Triglycerides, HDL-C, apo A-I, apo B, oxLDL, LCAT, enzyme that catalyzes serum cholesterol esterification, PON-1 activity, and in vitro transfers of cholesterol, triglycerides and phospholipids to HDL, important steps in HDL metabolism, were equal. IL-8 was higher in III/IV (*p* = 0.025), but TNFα, IL-1β, IL-6 and MCP-1 were equal. BNP was negatively correlated with CETP (*r* = − 0.294; *p* = 0.042) and positively correlated with IL-8 (*r* = 0.299; *p* = 0.039).

**Conclusions:**

Our results disclosed the relationship between CETP levels and HF severity, by comparing two HF groups and by correlation analysis. Lower CETP levels may be a marker of HF aggravation and possibly of worse prognosis. Practical applications of this initial finding, as the issue whether CETP could be protective against HF aggravation, should be explored in larger experimental and clinical studies.

## Background

Heart failure (HF) is the common final stage of several diseases of the heart, such as coronary artery disease, cardiomyopathies and systemic arterial hypertension, among others. In 2014, estimated 26 million subjects worldwide were suffering from HF, with 1-year mortality 20–30% and 5-year mortality 45–60%, roughly equal to the overall mortality rates of the malignant diseases [1]. The prevalence of HF is steadily growing as a consequence of the increasing percentage of aged individuals in the general population. Moreover, the improvements in the management of patients with acute cardiovascular diseases also account for the increasing rates of surviving subjects with chronic cardiomyopathy that subsequently develop HF [2].

In HF, the volume or pressure overload stressing the myocardial wall activates the genes coding for brain natriuretic peptide (BNP) in the cardiomyocytes [3]. BNP serum concentration that is very low in healthy individuals rises proportionally to the HF severity and is a chief biochemical parameter in HF classification criteria [4].

HF courses with a chronic inflammatory process and pro-inflammatory cytokines such as tumor necrosis factor α (TNFα), interleukin (IL) -1, IL-6 and IL-8 are increased in those patients [5]. The metabolism of plasma lipoproteins is also altered in HF and changes in LDL-cholesterol (LDL-C), HDL-cholesterol (HDL-C) and triglycerides have been often described [6–9]. Whether or not the intensity of changes in those inflammatory or lipid parameters are related with the severity or with the progression of HF has not yet been established.

Lipoprotein classes continuously exchange their constituent lipids, such as cholesterol in both unesterified and esterified forms, phospholipids and triglycerides. Cholesteryl ester transfer protein (CETP) and phospholipid transfer protein (PLTP) facilitate lipid transfers among lipoprotein classes. CETP is a hydrophobic glycosylated protein [10] that circulates in the plasma associated principally to the HDL fraction; and promotes the transfers of esterified cholesterol and triglycerides [11].

Unesterified cholesterol transferred to HDL undergoes esterification catalyzed by lecithin-cholesterol acyl transferase (LCAT), a reaction that occurs mostly at the HDL fraction. LCAT uses apolipoprotein (apo) A-I, the main HDL apo, as co-factor. Those lipid transfers may affect the composition and metabolism of the HDL fraction [12]. The association of in vitro lipid transfer and presence of coronary artery disease was already documented [13] but the status of this important metabolic process was not yet explored in HF.

The aim of this study was to test the hypothesis of whether the severity of HF is associated with alterations in parameters related with the HDL functional aspects, such as rates of lipid transfer to HDL, CETP and LCAT concentration, as well as the activity of paraoxonase-1 (PON-1), an anti-oxidant enzyme associated to the HDL fraction. A correlation study was performed between the HDL metabolism-related parameters and the BNP values. Plasma lipids and apolipoproteins, as well as inflammatory markers, were also included in the analysis.

## Methods

### Study subjects

Forty-eight patients with HF were selected from the Outpatient Clinics of the Heart Institute at the University of São Paulo Medical School and Santa Marcelina Hospital, both in the city of São Paulo, Brazil.

Table 1 shows the physical characteristics, clinical data and medications used by the HF participant patients. Twenty-five patients had class I and II (group HF I/II), the milder forms of the disease, and 23 had class III and IV (group HF III/IV), the most severe forms. The functional classification adopted was that of the New York Association for Heart Failure (NYHA).

**Table 1:** Physical characteristics, clinical data, etiology of the heart failure (HF) and medications used by patients with class I/II and III/IV heart failure (HF)

	HF I/II (*n* = 25)	HF III/IV (*n* = 23)	*p*-value
Age (years)	63 ± 13	66 ± 11	0.359
Gender, n (%)			0.407
Male	16 (64.0)	13 (56.5)	
Female	9 (36.0)	10 (43.5)	
BMI (Kg/m^2^)	28 ± 5	24 ± 4	***0.041***
Coronary artery disease, n (%)	14 (56.0)	7 (30.4)	0.067
Ischemic heart disease, n (%)	9 (36.0)	4 (17.4)	0.130
Hypertension, n (%)	16 (64.0)	15 (65.2)	0.585
Diabetes mellitus, n (%)	7 (28.0)	4 (17.4)	0.300
Hyperlipidemia, n (%)	10 (40.0)	3 (13.0)	***0.036***
HF etiology, n (%)			
Hypertension	7 (28.0)	8 (34.8)	0.422
Ischemic	9 (36.0)	4 (17.4)	0.130
Dilated cardiomyopathy	6 (24.0)	7 (30.4)	0.430
Myocarditis	3 (12.0)	2 (8.7)	0.541
Idiopathic	0 (0)	2 (8.7)	0.224
Medication, n (%)			
ACE inhibitors	20 (80.0)	21 (91.3)	0.244
Beta blockers	13 (52.0)	17 (73.9)	0.102
Aldosterone antagonists	4 (16.0)	22 (95.7)	***< 0.001***
Diuretics	16 (64.0)	15 (65.2)	0.585
Antiplatelet	13 (52.0)	7 (30.4)	0.111
Anticoagulants	1 (4.0)	3 (13.0)	0.273
Antiarrhythmic	7 (28.0)	0 (0.0)	***0.007***
Metformin	9 (36.0)	6 (26.1)	0.335
Statin^a^	10 (40.0)	3 (13.0)	***0.036***

Frequencies distributions are expressed in number of cases (n) and percentage (%). Age and BMI are expressed as mean ± standard deviation. *ACE* angiotensin-converting enzyme, *BMI* body mass index

^a^In patients under statin use, the treatment was discontinued for 30 days before they were studied

The inclusion criterion was stable systolic HF left ventricular ejection fraction < 40%, determined by echocardiography. The statin treatment of the patients was discontinued for 30 days before they participated in this study. Exclusion criteria for all groups were severe arterial hypertension, renal and hepatic failure, hypothyroidism and recent surgery. The study protocol was in accordance with the Declaration of Helsinki and was approved by the Ethics Committees of both institutions and an informed consent term was signed by all participants.

### Biochemical assay

The blood samples were collected after 12-h fasting. Commercial enzymatic colorimetric methods were used to determine triglycerides and total cholesterol (Roche, Basel, Switzerland). HDL-C was measured by the same method used for total cholesterol after apo B containing-lipoprotein precipitation. LDL-C was calculated by the Friedewald equation [14] and non-HDL-C was determined by the equation: total cholesterol minus HDL-C. Apo A-I and B were measured by rate turbidimetry (Roche, Basel, Switzerland). BNP was measured by the immunoassay (Abcam, Cambridge, UK).

### Determination of CETP, LCAT and oxidized LDL concentration

Serum concentrations of CETP and LCAT were determined by immunoassay (ALPCO Diagnostics, Salem, USA). Concentration of oxidized LDL (oxLDL) also measured by immunoassay, using a monoclonal antibody 4E6 kit (Mercodia, Uppsala, Sweden).

### Determination of PON-1 activity

PON-1 activity was measured by adding 500 μL of 0.1 M Tris-HCl buffer (pH 8.05) containing 2 mmol/L CaCl_2_ and 1.1 mmol/L paraoxon (Sigma Aldrich, St. Louis, USA) to a volume of 25 μl of each serum patient [15]. Then, 200 μL of the mixture from each sample was pipetted into a 96-well plate. The reading was done at 405 nm at 37 °C.

### Preparation of the artificial lipid nanoparticle

The lipid donor nanoparticle was prepared from a lipid mixture described by Maranhão et al. [16]. In a vial, 40 mg cholesteryl oleate, 20 mg egg phosphatidylcholine, 1 mg triolein and 0.5 mg cholesterol, purchased from Sigma Aldrich, were mixed. Trace amounts of glycerol tri [9,10(n)-^3^H] oleate and 4-^14^C-cholesterol or [1α,2α(n)-^3^H]-cholesteryl oleate and L-3-phosphatidylcholine,1-stearoyl-2-[1-^14^C] arachidonyl (Amersham, Little Chalfont, UK) were added to the initial solution. The lipids were emulsified by prolonged ultrasonic irradiation in aqueous media and a two-step ultracentrifugation of the crude emulsion with density adjustment by addition of KBr to obtain the nanoparticle. The nanoparticle fraction was dialyzed against a 0.9% NaCl solution.

### Lipid transfer from the donor nanoparticle to HDL

The in vitro assay of lipid transfer from the lipid nanoparticle to HDL was previously described by Lo Prete et al. [17]. An aliquot of 200 μL of the plasma with EDTA was incubated with 50 μL of the nanoparticle labeled with ^14^C-cholesterol and ^3^H-triglycerides or with ^3^H-cholesteryl esters and ^14^C-phospholipids. After 1 h incubation at 37 °C, 250 μL of dextran sulfate/MgCl_2_ was added as precipitation reagent. The mixture was shook for 30 s, centrifuged for 10 min at 3000 g. Aliquots of 250 μL of the supernatant were added in 5 mL of scintillation solution (Packard BioScience, Groeningen, Netherlands) and the radioactivity was measured in liquid scintillation analyzer (Packard). The transfer of each lipid from the nanoparticle to plasma HDL was expressed as % of the total incubated radioactivity.

### Serum concentrations of inflammatory and metabolic markers

Serum concentrations of IL-1β, IL-6, IL-8, TNFα, MCP-1 (monocyte chemotactic protein), insulin and leptin were determined simultaneously by the Human Adipokine Magnetic Bead Panel 2 Kit HADK2MAG-61 K (EMD Millipore, Billerica, USA), according to the manufacturer’s instructions. The concentrations were calculated from best-fit standard curves generated from calibrators for each analyte.

### Statistical analyses

Statistical analyzes were conducted using SPSS 19.0 statistical software (SPSS® Advanced Statistics, IBM Corporation, Illinois, USA). Categorical data, i.e.,HF etiology, gender, presence of coronary artery disease, ischemic heart disease, diabetes mellitus, hypertension and hyperlipidemia, and use of medications were analyzed using the chi-square test (χ2). The distribution of the continuous data was evaluated by the Shapiro-Wilk test and the data were compared using the unpaired Student’s *t* test or the Mann-Whitney *U* test, depending on the Gaussian distribution. For multivariate analysis, all parameters were adjusted for BMI and presence of hyperlipidemia and coronary artery disease. These analyses were performed using generalized linear models with normal or gamma distribution and identity or logarithmic link function. Correlation analyses were assessed using Spearman’s rho correlation test. In all analyses, parameters were considered significantly different when *p* < 0.05.

## Results

### Plasma biochemical parameters and lipid transfers to HDL

Table 2 shows serum biochemistry and in vitro lipid transfers to HDL data. Body mass index (BMI) was lower in the HF III/IV (*p* = 0.041). As expected, BNP concentration was higher in HF III/IV than in HF I/II group (*p* < 0.001). Leptin levels were lower in the HF III/IV (*p* = 0.004). Glycemia was higher in the HF III/IV group (*p* = 0.008), but insulin levels were not different.

**Table 2:** Serum biochemistry and in vitro lipid transfers to HDL data of the group of patients with class I/II and III/IV heart failure (HF)

	HF I/II (*n* = 25)	HF III/IV (*n* = 23)	*p*-value	*p*-value ^b^
BNP (pg/mL)	265 ± 77 (144–401)	1066 ± 621 (531–3350)	***< 0.001*** ^a^	***< 0.001*** ^c^
Glucose (mg/dL)	96 ± 14 (78–125)	115 ± 33 (79–231)	***0.008*** ^a^	***0.002*** ^c^
Insulin (pg/mL)	362 ± 246 (48–887)	532 ± 795 (37–3426)	0.757 ^a^	0.061 ^c^
Leptin (pg/mL)	34,000 ± 35,082 (2229–118,501)	16,286 ± 25,517 (440–96,772)	***0.004*** ^a^	0.090 ^c^
Triglycerides (mg/dL)	119 ± 33 (61–193)	120 ± 50 (55–278)	0.726 ^a^	0.697 ^c^
Cholesterol (mg/dL)				
Total	192 ± 38 (105–274)	170 ± 37 (105–269)	***0.049***	***0.031***
LDL	143 ± 35 (70–228)	118 ± 38 (52–222)	***0.023***	***0.013***
non-HDL	167 ± 39 (82–254)	141 ± 39 (76–249)	***0.029***	***0.016***
HDL	25 ± 6 (17–41)	28 ± 11 (17–65)	0.351 ^a^	0.252 ^c^
Apolipoproteins (mg/dL)				
A-I	126 ± 23 (80–162)	133 ± 31 (96–217)	0.388	0.392 ^c^
B	92 ± 27 (57–161)	81 ± 35 (25–165)	0.230	0.175
Oxidized LDL (U/L)	51.55 ± 22.22 (17.20–101.20)	40.79 ± 20.72 (11.70–78.50)	0.089	0.070
PON-1 (nmol/min/mL)	111.96 ± 62.66 (21.60–245.50)	132.91 ± 81.07 (29.70–315.10)	0.427 ^a^	0.147 ^c^
LCAT (μg/mL)	8.43 ± 2.28 (4.00–11.60)	8.26 ± 3.32 (3.30–15.80)	0.842	0.498
CETP (μg/mL)	3.73 ± 1.30 (1.33–5.52)	2.77 ± 1.30 (0.81–5.26)	***0.013***	***0.002***
Lipid Transfers (%)				
Esterified cholesterol	5.97 ± 0.82 (4.07–7.60)	5.44 ± 1.76 (2.61–9.44)	0.192	0.275
Unesterified cholesterol	6.73 ± 1.12 (4.33–9.89)	6.29 ± 2.05 (3.07–9.78)	0.363	0.455
Phospholipids	19.91 ± 1.12 (17.33–22.59)	19.05 ± 2.50 (14.16–23.85)	0.139	0.122
Triglycerides	8.45 ± 1.31 (6.28–11.69)	8.07 ± 2.27 (4.31–11.89)	0.504	0.900

Data are expressed as mean ± standard deviation (min-max). *BNP* brain natriuretic peptide, *HDL* high-density lipoprotein, *LDL* low-density lipoprotein, *PON-1* paraoxonase 1, *LCAT* lecithin-cholesterol acyltransferase, *CETP* cholesterol ester transfer protein

^a^ Non-parametric Mann-Whitney U-test

^b^ All models were adjusted by BMI, hyperlipidemia and coronary artery disease; The analysis were performed using generalized linear models with normal distribution and identity link function

^c^Generalized linear models with gamma distribution and identity link function

Concentrations of total cholesterol (*p* = 0.049), LDL-C (*p* = 0.023) and non-HDL-C (*p* = 0.029) were lower in the HF III/IV group. Regarding the concentrations of triglycerides, HDL-C, apo A-I, apo B and oxLDL, no differences were found between the two groups (Table 2).

CETP concentration was lower in HF III/IV than in HF I/II group (*p* = 0.013), but LCAT concentration and PON-1 activity were not different between groups (Table 2). Transfer of the four lipids to HDL is shown on Table 2. Comparing HF III/IV with HF I/II, there were no differences in transfers of esterified and unesterified cholesterol, phospholipids and triglycerides.

The results described above did not change when all the parameters were adjusted for BMI and presence of coronary artery disease and hyperlipidemia, except for the leptin data. After adjustment for those parameters, leptin concentration became not different between the HF I/II and HF III/IV groups (Table 2).

### Inflammatory markers

Table 3 shows the serum concentrations of inflammatory markers. IL-8 was higher in HF III/IV group (*p* = 0.025), but the concentrations of TNFα, IL-1β, IL-6 and MCP-1 were not different between the two groups. Even after adjusting all these parameters for BMI, coronary artery disease and hyperlipidemia, the above described results did not change (Table 3).

**Table 3:** Inflammatory cytokines concentrations data (pg/mL) of the group of patients with class I/II and III/IV heart failure (HF)

	HF I/II (*n* = 25)	HF III/IV (*n* = 23)	*p*-value	*p*-value ^b^
TNFα	4.45 ± 2.44 (1.29–9.68)	4.93 ± 3.65 (1.22–18.61)	0.733 ^a^	0.546 ^c^
IL-1β	1.10 ± 3.25 (0.19–16.64)	0.52 ± 0.36 (0.19–1.88)	0.611 ^a^	0.280 ^c^
IL-6	9.45 ± 13.04 (0.25–45.00)	13.61 ± 15.02 (0.29–60.86)	0.165 ^a^	0.166 ^d^
IL-8	5.30 ± 3.42 (1.41–14.86)	9.82 ± 8.35 (0.62–31.94)	***0.025*** ^a^	***0.027*** ^***c***^
MCP-1	140.17 ± 45.87 (67–233)	159.67 ± 58.22 (65–258)	0.206	0.220

Values are expressed as mean ± standard deviation (min-max). *TNFα* tumor necrosis factor α, *IL* interleukin, *MCP-1* monocyte chemotactic protein

^a^ Non-parametric Mann-Whitney U-test

^b^ All models were adjusted by BMI, hyperlipidemia and coronary artery disease; The analysis were performed using generalized linear models with normal distribution and identity link function

^c^ Generalized linear models with gamma distribution and identity link function

^d^ Generalized linear models with normal distribution and logarithmic link function

### Correlation analyses

**Fig. 1:**
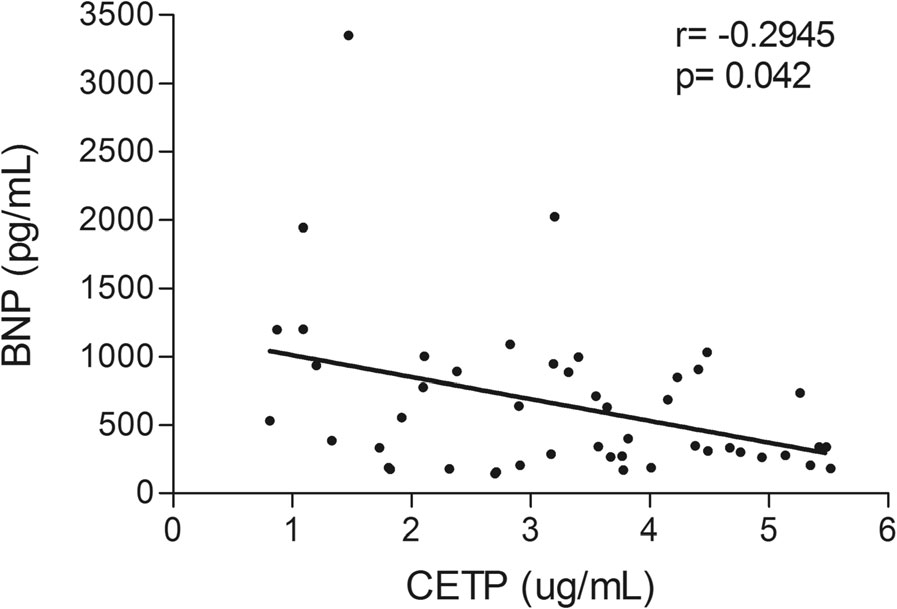
Correlation between CETP (cholesterol ester transfer protein) and BNP (brain natiuretic protein) in patients with heart failure

Table 4 shows the results of the correlation study performed by univariate analysis of the HF class, concentrations of BNP, triglycerides, LDL-C, HDL-C, CETP, and rates of transfer of the four lipids plotted against the plasma concentration of glucose, insulin, leptin, triglycerides, total cholesterol, HDL-C, non-HDL-C and LDL-C, apo A-I, apo B, oxLDL, LCAT, CETP, inflammatory cytokines, and PON-1 activity, as well as the values of the lipid transfer in the two groups studied. Figure 1 shows the correlation of BNP levels against CETP concentration.

**Table 4:** Correlation analyses of HF class, BNP values, serum biochemical data, in vitro lipid transfers to HDL and inflammatory cytokines in patients with class I/II and III/IV heart failure (HF)

Parameters	HF class	BNP	Triglycerides	LDL-C	HDL-C	CETP	Esterified-C Transfer	Unesterified-C Transfer	Phospholipids Transfer	Triglycerides Transfer
BNP	***0.8655*** ^*** θ***^	__	− 0.0796	− 0.2460	0.0345	− 0.2945 *	− 0.2400	0.0150	− 0.2372	− 0.0292
BMI	− 0.2691	***− 0.3109*** *	− 0.0073	− 0.1885	0.1419	0.0346	0.2257	0.1757	0.0930	0.3225 *
Glucose	***0.3939*** ^**†**^	0.3136 *	***0.3933*** ^**†**^	−0.1421	***0.4144*** ^**†**^	− 0.2327	− 0.0716	− 0.0024	− 0.0512	0.0629
Insulin	− 0.0452	− 0.1081	− 0.0815	−0.0936	0.0255	0.1348	0.0925	0.1807	−0.1067	0.2830
Leptin	***−0.4199*** ^**†**^	−0.3649 *	− 0.1362	0.2221	− 0.2751	0.1768	0.1152	0.1407	0.1519	0.1000
Triglycerides	−0.0512	− 0.0796	__	0.2688	***0.6760*** ^**θ**^	0.1490	0.0361	−0.0504	0.0522	0.0962
Total-C	−0.3147 *	− 0.2307	***0.6299*** ^**θ**^	***0.9100*** ^**θ**^	−0.0423	0.2688	0.1008	0.0199	0.1352	0.0488
HDL-C	0.1359	0.0345	***0.6760*** ^**θ**^	***−0.4081*** ^**†**^	__	−0.0326	0.0281	− 0.0094	− 0.00003	0.1765
non-HDL-C	−0.3252 *	− 0.2211	***0.4377*** ^**†**^	***0.9802*** ^**θ**^	−0.2484	0.2422	0.0657	0.0315	0.0986	0.0269
LDL-C	−0.3598 *	−0.2460	0.2688	__	***−0.4081*** ^**†**^	0.2365	0.0712	0.0278	0.1013	0.0095
Apo A-I	0.0813	0.1029	0.1962	−0.1002	0.2204	−0.1409	− 0.0284	− 0.0175	0.1121	− 0.0044
Apo B	− 0.2304	− 0.2665	0.3524 *	0.2253	0.0952	−0.0315	0.0990	0.0131	0.1420	0.0266
OxLDL	−0.2755	−0.2673	0.1361	−0.0006	0.0811	0.3569 *	0.0148	−0.0749	0.0895	−0.0158
PON-1	0.1159	0.0773	0.0095	0.1349	−0.1210	−0.2301	− 0.0472	0.0216	0.0210	−0.1502
LCAT	−0.0557	−0.1549	0.1886	0.1560	−0.0141	0.0720	−0.1230	−0.2396	− 0.0466	−0.1247
CETP	−0.3537 *	−0.2945 *	0.1490	0.2365	−0.0326	__	0.0618	−0.1165	−0.0510	− 0.0636
Esterified-C transfer	−0.2695	− 0.2400	0.0361	0.0712	0.0280	0.0618	__	***0.5968*** ^***#***^	***0.8610*** ^***#***^	***0.5811*** ^***#***^
Unesterified-C transfer	−0.0963	0.0150	−0.0503	0.2783	−0.0094	− 0.1165	***0.5968*** ^***#***^	__	***0.4816*** ^**†**^	***0.8100*** ^***#***^
Phospholipids transfer	−0.2484	−0.2372	0.0521	0.1013	−0.00003	−0.0510	***0.8610*** ^***#***^	***0.4816*** ^**†**^	__	***0.4706*** ^**†**^
Triglycerides transfer	−0.0805	−0.0292	0.0962	0.0095	0.1765	−0.0636	***0.5811*** ^***#***^	***0.8100*** ^***#***^	***0.4706*** ^**†**^	__
TNFα	0.0497	0.0627	***−0.4100*** ^***†***^	−0.0669	− 0.2447	0.0470	− 0.0249	0.2043	− 0.0986	0.0873
IL-1β	0.0741	0.0890	−0.1696	−0.1493	− 0.1066	0.0361	− 0.1165	−0.0419	− 0.2945 *	−0.1987
IL-6	0.2095	0.1344	***−0.3807*** ^**†**^	−0.3123 *	− 0.1766	−0.0466	− 0.1994	0.0334	− 0.2596	−0.0107
IL-8	0.3267 *	0.2995 *	−0.2942 *	−0.3227 *	0.0277	−0.2796	− 0.0582	0.0671	− 0.0851	0.1051
MCP-1	0.1686	0.0935	−0.2904 *	−0.0964	− 0.1427	0.2334	− 0.2720	−0.1403	− 0.3189 *	−0.0526
HF class	__	***0.8655*** ^***θ***^	−0.0512	***−0.3598*** ^*^	0.1359	−0.3537 *	− 0.2695	−0.0963	− 0.2484	−0.0805

*BNP* brain natriuretic peptide, *BMI* body mass index, *-C* cholesterol, *HDL-C* high-density lipoprotein cholesterol, *LDL-C* low-density lipoprotein cholesterol, *Apo* apolipoprotein, *OxLDL* oxidized LDL, *PON-1* paraoxonase 1, *LCAT* lecithin-cholesterol acyltransferase, *CETP* cholesterol ester transfer protein, *TNFα* tumor necrosis factor α, *IL* interleukin, *MCP-1* monocyte chemotactic protein. *p < 0.05. ^†^*p* < 0.01. ^#^*p* < 0.001. ^**θ**^*p* < 0.0001

As expected, the BNP concentration was positively correlated with HF class (*r* = 0.865; *p* < 0.0001). BNP values were also positively correlated with IL-8 concentration (*r* = 0.299; *p* = 0.039). BNP was negatively correlated with BMI (*r* = − 0.311; *p* = 0.031) and CETP concentration (*r* = − 0.294; *p* = 0.042). CETP concentration was positively correlated with oxLDL (*r* = 0.357; *p* = 0.013) and inversely correlated with HF class (*r* = − 0.354; *p* = 0.014).

HF class was positively correlated with IL-8 concentration (*r* = 0.327; *p* = 0.023) and negatively correlated with CETP (*r* = − 0.354; *p* = 0.014), total cholesterol (*r* = − 0.315; *p* = 0.029), LDL-C (*r* = − 0.360; *p* = 0.012), non-HDL-C (*r* = − 0.325; *p* = 0.024) concentration.

HDL-C was positively correlated with triglycerides concentration (*r* = 0.676; *p* < 0.0001), and negatively correlated with LDL-C (*r* = − 0.408; *p* = 0.004).

LDL-C was positively correlated with total cholesterol (*r* = 0.910; *p* < 0.0001) and non-HDL-C (*r* = 0.980; *p* < 0.0001), and negatively correlated with HF class (r = − 0.360; *p* = 0.0120), and with concentrations of IL-6 (*r* = − 0.312; *p* = 0.037) and IL-8 (*r* = − 0.323; *p* = 0.025).

Triglyceride concentration was positively correlated with total cholesterol (*r* = 0.630; p < 0.0001), non-HDL-C (*r* = 0.438; *p* = 0.002) and apo B (*r* = 0.352; p = 0.014) concentration. Triglyceride concentration was negatively correlated with inflammatory markers: IL-6 (*r* = − 0.381; *p* = 0.010), IL-8 (*r* = − 0.294; *p* = 0.042), TNFα (*r* = − 0.410; p = 0.004) and MCP-1 (*r* = − 0.290; *p* = 0.045).

Regarding the correlations with the values of lipid transfer, the only correlations found were negative correlations of phospholipid transfer with the concentration of MCP-1 (*r* = − 0.319; *p* = 0.027) and IL-1β (*r* = − 0.294; p = 0.042).

## Discussion

Among the classical plasma lipid parameters, it has been reported that LDL-C is reduced in HF and associated to poorer prognosis [9]. Our current results show that, in fact, in patients with the most severe HF, classes III/IV, LDL-C was lower than in the milder forms of the disease. Here, the non-HDL-C levels, that represent the cholesterol content of all apo B containing-lipoproteins and not only LDL-C, were also lower in the severe HF forms compared with the patients with the milder forms. Low HDL-C has also been associated with the presence of HF [18]. HF patients with higher HDL-C [6, 7, 19] and higher apo A-I concentration [20, 21] have longer survival rates, but in regard to this lipoprotein fraction, no differences between HF severe and milder forms were found in our study.

The main novel finding of this study was that patients with the severe forms of HF (classes III/IV) compared with the milder forms of the disease (classes I/II) had lower CETP concentration. The involvement of CETP in atherosclerosis, whether this protein is beneficial or pro-atherogenic, is largely controversial. CETP inhibitors, that pronouncedly increase anti-atherogenic HDL-C, have failed to decrease the frequency of the clinical manifestations of coronary artery disease [22]. This is the first study to measure CETP in HF and the finding that CETP is lower in the III/IV than I/II group was further supported by the negative correlation between CETP concentration and BNP values. Beyond the role in the lipid exchanges among lipoprotein classes, CETP has also postulated roles in inflammatory processes and in the immunological defenses of the organism [23, 24], which enlightens the importance of this finding.

It was worthwhile to point out that lower CETP concentration in the III/IV patients was not followed by accordingly lower transfer of lipids to HDL in that group. As CETP facilitates the transfers of cholesteryl esters and triglycerides, it would be expected that transfer of these lipids would be diminished in the severe form of the disease which did not occur: the transfer of all four lipids was not different between severe and milder HF patients. In fact, lipid transfer does not depend only on CETP action, but also on many other factors such as the concentration and composition of the lipoprotein classes and the activity of several enzymes that modulate the plasma lipid metabolism.

The meaning of the positive correlation found here between CETP and oxLDL is difficult to ascertain. At any rate, CETP is largely involved with the lipid fluxes in the plasma compartment and the generation of oxLDL depends on the LDL kinetics in the plasma, so that it is possible that this correlation could eventually bear physiological implications.

LCAT concentration that had not previously been evaluated in HF was not different between severe and milder HF forms. LCAT catalyzes the cholesterol esterification in the plasma, using apo A-I, the main apolipoprotein present in the HDL composition, as co-factor. Esterification of cholesterol is important for the plasma cholesterol homeostasis. LCAT promotes the cholesterol reverse transport and the stabilization of cholesterol in the plasma compartment [25].

The exacerbation of the inflammatory status associated with HF, that can be a factor of aggravation of the disease [5], was also documented here by the higher concentration of IL-8, a pro-inflammatory cytokine in the severe compared to the milder HF patients. This observation was strengthened by our current finding of a positive correlation between IL-8 values and those of BNP. Noteworthy, patients with worse HF status and lower left ventricular function had showed not only higher IL-8 but also higher IL-6 and TNFα [26–29]. Here, in respect to IL-6 and TNFα, those differences between the milder and the severe forms were not statistically significant, possibly due to the small population size and the large data dispersion of those parameters.

Interestingly, inverse correlations were found between LDL-C and triglycerides versus pro-inflammatory cytokines IL-6 and IL-8. In view that low LDL-C has been considered an aggravating factor for HF together with intensified inflammation; it is possible that there should be a link between lipid metabolism and the inflammatory process driving disease progression.

The multivariate analysis of the data as adjusted by BMI, coronary artery disease and hyperlipidemia did not substantially change the results of the comparisons obtained by univariate analysis. However, the multivariate analysis did not confirm the finding that leptin levels were higher in HF I/II than in HF III/IV. In fact, it is known that leptin is positively correlated with BMI; HF aggravation, as in III/IV patients, leads to weight loss. On the other hand, the two groups differed as to the % of patients with hyperlipidemia, and tended to be different, although not statistically significant, as to the % of coronary artery disease presence. The multivariate analysis, however, confirmed that the findings that CETP concentration was lower and IL-8 was higher in HF III/IV were independent of % hyperlipidemia or % coronary artery disease. Incidentally, the fact that statin use was more frequent in HF I/II was most likely related to the greater presence of hyperlipidemia in this group.

## Conclusions

This study suggests CETP as a possible new player among the factors that may drive the aggravation of HF. This assumption is supported by the lower levels of this protein in the most severe HF classes and by the inverse correlation between CETP and BNP concentrations. As several CETP inhibitors have been tested in large clinical trials to increase HDL-C and thereby reduce cardiovascular events, this novel finding of low CETP related with HF progression may bear potentially important therapeutic implications.

## Abbreviations

ACE: Angiotensin-converting enzyme
apo: Apolipoprotein
BMI: Body mass index
BNP: Brain natriuretic peptide
CETP: Cholesterol ester transfer protein
HDL-C: High-density lipoprotein cholesterol
HF: Heart failure
IL: Interleukin
LCAT: Lecithin-cholesterol acyltransferase
LDL-C: Low-density lipoprotein cholesterol
MCP-1: Monocyte chemotactic protein
NYHA: New York Association for Heart Failure
OxLDL: Oxidized LDL
PLTP: Phospholipid transfer protein
PON-1: Paraoxonase 1
TNFα: Tumor necrosis factor α
